# Flow characteristics of open channels based on patch distribution of partially discontinuous rigid combined vegetation

**DOI:** 10.3389/fpls.2022.976646

**Published:** 2022-10-11

**Authors:** Jingzhou Zhang, Shengtang Zhang, Chuantao Wang, Wenjun Wang, Lijun Ma

**Affiliations:** College of Earth Science and Engineering, Shandong University of Science and Technology, Qingdao, China

**Keywords:** open channel flow, combined distribution, discrete distribution, patch vegetation, numerical simulation

## Abstract

To clarify the flow characteristics of open channels under the combined distribution of vegetation in a patch, this study used the computational fluid dynamics tool FLUENT and the Reynolds stress model to design four combined and four discrete distribution modes under two different inundation states (submerged and non-submerged). The flow characteristics of longitudinally discontinuous rigid vegetation patches occupying half the width of the channel were numerically simulated. The numerical model is verified by indoor open channel flume experiments, and the obtained model data is in good agreement with the measured data. The results showed that: 1) The diameter of vegetation is an important factor affecting the wake structure. Under the submerged condition. 2)The submerged state, distribution pattern and combination form of vegetation are important factors that affect the distribution of flow velocity and change the structure of water flow. That is, the influence of vegetation distribution pattern on flow velocity and turbulence intensity under submerged condition is significantly weaker than that under non-submerged condition, and the flow velocity in non-vegetation area is significantly higher than that in vegetation area. The increase in the combined vegetation comprehensive stem thickness and the discrete degree resulted in an increase in the difference in flow velocity and turbulence intensity. 3) As the water flowed downstream, the flow velocity along the vegetated area continuously decreased, while it increased continuously along the non-vegetated area, and the difference in flow velocity between the two areas became more apparent. 4) The inundation state and combination characteristics of vegetation were important factors affecting the Reynolds stress of the channel location in the patch area.

## 1 Introduction

In recent years, the construction of ecological river courses has been vigorously promoted around the world and the coverage of vegetation in floodplains, and even the main channel, has been greatly improved. Vegetation is not only an important part of the river ecosystem, but also an important factor to consider during ecological river construction (Schulz et al., 2003; Cotton et al., 2006; Fathi-Moghadam et al., 2011; Curran and Hession, 2013; Zdankus et al., 2016). Vegetation can inhibit sediment suspension, purify the water environment, prevent water flow from eroding riverbanks, and have great practical value in maintaining the ecological function stability of river systems (Kemp et al., 2000; Wu et al., 2007; Nept, 2012; Aberle and Järvelä, 2013; Boothroyd et al., 2017). However, the existence of vegetation changes the flow characteristics of the channel, affects the flow velocity and turbulence characteristics, and changes the relationship between the storage and discharge of the channel, thereby changing the process of river confluence (Zhang and Su, 2008; Rominger and Nepf, 2011; Velísková et al., 2017), which has a non-negligible impact on the advancement of river flow, especially flood runoff in flood season (Mulahasan and Stoesser, 2017; Nosrati et al., 2022).

To date, there have been many achievements and advancements in research on the hydrodynamics of river vegetation. The distribution forms of vegetation in river channels are varied. In previous studies on river flow covered by vegetation, vegetation was mainly distributed uniformly or regularly throughout the whole test area. Nadaoka and Yagi (1998) used a Large Eddy Simulation (LES) model to study the turbulent flow characteristics of open channels with vegetation. Liu et al. (2021) studied the potential mechanism of morphological resistance of emergent vegetation under different vegetation densities and patterns by combining experimental and numerical simulation and concluded that the morphological resistance of vegetation is closely related to eddy current structure near vegetation. Tang et al. (2020) studied the velocity and turbulence characteristics of two kinds of flexible vegetation with different densities using an open channel test and concluded that the maximum velocity gradient and shear stress gradient appeared near the top of canopy. Using uniform grass cover, Yang et al. (2017) found that the resistance coefficient increased significantly with the increase of vegetation diameter, which effectively extended the retention time of concentrated flow on the slope, thus reducing soil erosion. This kind of model setup is relatively simple and is mainly used to study the influence of overall vegetation distribution on the hydraulic characteristics of open channels (Tanino and Nepf, 2008; Cheng et al., 2019), however, the flow characteristics caused by local vegetation distribution in river channels cannot be simply obtained from the overall vegetation flow characteristics.

In recent years, the water flow with partial vegetation distribution in open channels has received increasing attention because wetlands and floodplains have partial vegetation distribution, and the distribution of vegetation along riverbanks is a common feature of natural rivers and artificial ecological channels (Tooth and Nanson, 2000; Jang and Shimizu, 2007; White and Nepf, 2008; Truong and Uijttewaal, 2019). White and Nepf (2007) studied the flow structure and energy exchange in open channels distributed along riverbanks by some vegetation, and found that the shear layer at the junction between vegetated and non-vegetated domains has a double-layered structure. Tang et al. (2021) arranged two kinds of rigid vegetation with different heights on the side of the open channel to conduct an experimental study on the turbulent characteristics of river flow. The experimental results showed that the flow velocity and Reynolds stress increased sharply at the top of the vegetation, and that there was a strong mixed layer near the top of the vegetation. In addition, there was a strong shear layer between the non-vegetated area and the vegetated area, indicating that vegetation reduces flow velocity. Huai et al. (2019) laid continuous artificial vegetation on one side of an open channel to study the energy exchange and water turbulence characteristics between the vegetated and non-vegetated areas to determine whether artificial vegetation could be used to increase the water depth of natural rivers and improve navigation. Caroppi et al. (2022) focused on the effects of the flexible-induced reconfiguration of vegetation leaves on the wake and flow around the vegetation zone, on one side of a river channel, through experiments. They found that leaves increased vegetation resistance by 3.0–4.4 times, and the streamlined and reconstructed leaves reduced vegetation resistance by 60%.

However, for natural river channels, vegetation tends to be distributed in longitudinal discontinuous along riverbanks due to seasonal and temporal changes (Tanaka et al., 2008; Tanaka and Ohmoto, 2015; Yang et al., 2015). In addition, under the resource limitation of oxygen and other nutrients, aquatic vegetation can spontaneously develop spatial inhomogeneity and self-organize to form vegetation patches (Bordeu et al., 2016; Ruiz-Reynés et al., 2017), so that riparian vegetation presents the spatial configuration of vegetation landscape with longitudinal discontinuous patches distribution. Previous studies have mostly focused on the overall and continuous vegetation distribution, and even experimental and numerical simulation studies of patch vegetation have primarily focused on a single vegetation patch (Takemura and Tanaka, 2007; Nicolle and Eames, 2011; Chang et al., 2017; Li et al., 2018; Tny et al., 2019) or the interaction between two patches (Vandenbruwaene et al., 2011; Meire et al., 2014; Ghani et al., 2019a). For example, Kazem et al. (2021a); Kazem et al. (2021b) studied the vortex structure in the channel of vegetation patch and the characteristics of turbulence gradually subsiding in the undeveloped area downstream of the patch by changing the patch size, and its research results showed that the presence of patches has a great influence on the flow structure inside and around the patches, and there are three different flow layers downstream of vegetation patches: wake layer, mixed layer and shear layer. However, studies on the patch distribution of partially discontinuous vegetation on open channel flow is not systematic enough, although this vegetation distribution pattern is more common in natural channels (Anjum and Tanaka, 2020; Li et al., 2020). In addition, most current studies use the uniform size of vegetation for generalized simulation processing, which does not conform to the natural situation of river vegetation (Sand Jensen and Madsen, 1992). Owing to the different vegetation species, growth cycles, and planting methods, the stem thickness and morphology of vegetation in river channels differ, resulting in a variety of river flow characteristics with different effects to that of uniform vegetation. At present, there are few studies on the influence of combined vegetation on open channel flow. Järvelä (2002) studied the flow-resistance coefficient using a combination of willow and sedge and concluded that the main vegetation biological characteristics that affect flow resistance include vegetation density, water-facing area of individual plants, vegetation stem thickness, and vegetation flexibility. However, the two types of vegetation belong to completely different species, and the research is mainly focused on qualitative research, and it is difficult to carry out quantitative research. Zhang et al. (2020) conducted overland flow scour experiments on vegetation with combinations of two different stem diameters, and found that the diameter of a plant has a power function relationship with the Darcy–Weisbach resistance coefficient. Under the same water depth, when the stem diameter of one plant remained constant, the flow resistance coefficient increased with the increase in the stem diameter of the other plant. Because the distribution characteristics of vegetation and the structure of overland flow are quite different from those of open channels, the research results cannot be completely applied to open channel flow. Therefore, the research on combined vegetation patches in open channels is very limited. Therefore, it is necessary to comprehensively consider the influence of partial discontinuous patch combination vegetation on river flow characteristics in order to properly replicate the riparian environment and provide an effective scientific basis for ecological river management and river restoration engineering design.

The purpose of this study is to simulate the three-dimensional flow structure of longitudinal discontinuous patch combination vegetation in a rectangular open channel. the aims are to: (1) determine the longitudinal distribution of streamwise velocity before and after the introduction of a single vegetation type with different stem thicknesses in the combined vegetation patches under different submerged states, (2) measure the effects of the combination forms and partial discontinuous distribution patterns of vegetation patches on the changes of streamwise and lateral flow velocity under different submergence conditions using a specific cross-section, (3) use the spatial distribution of transverse, longitudinal, and vertical velocity contours to analyze the distribution characteristics of flow velocity and energy exchange of open channels with partial discontinuous combined vegetation patches, (4) identify the influence of vegetation combination and discrete distribution characteristics on the vertical distribution of Reynolds stress under different submerged states using the specific position of the channel in the patch area, and to elucidate the spatial distribution inhomogeneity of the flow field under different vegetation distribution conditions, and (5) clarify the influence of vegetation combination and discrete distribution on water flow turbulence characteristics using the variation law of turbulent kinetic energy (TKE) in vegetated and non-vegetated areas under different submerged states in relation to the longitudinal distribution of TKE in a specific longitudinal section.

## 2 Materials and methods

### 2.1 Modelling setup and boundary conditions

**Figure 1:**
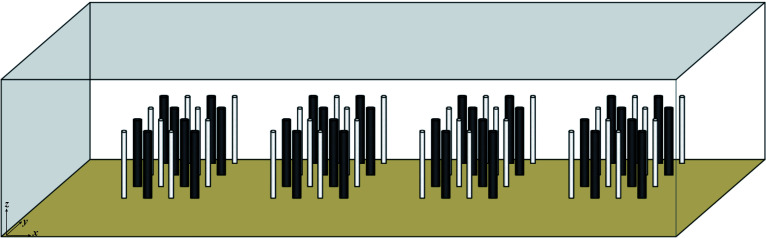
Three-dimensional view of numerical calculation area.

**Figure 2:**
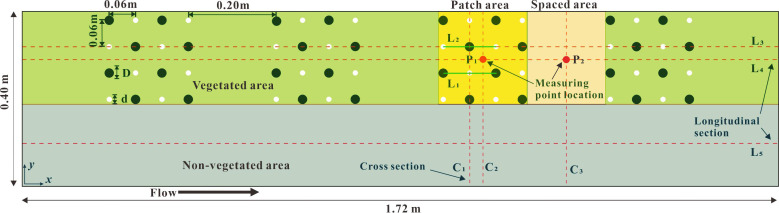
Schematic diagram of floor layout in numerical calculation area.

The fluid mechanics simulation of the open channel was carried out using the software FLUENT, and the Reynolds Stress Turbulence Model (RSM) was used to model the computational domain with a length of 1.72 m and a width of 0.4 m. The area was composed of rigid combined vegetation patches with a longitudinal discontinuous distribution. For the simulated vegetation, since the object of this study is the combined vegetation patches of different stem thicknesses, and the vegetation stems are usually cylindrical, so this study uses cylinders with different diameters to simulate vegetation. In addition, in order to enhance the simulation and experimental effects and improve the efficiency, most of the current studies also generalize the whole plant to an effective water blocking cylinder (Zhao and Huai, 2016; Anjum and Tanaka, 2020; Wang et al., 2021). In this study, a cylinder with a height of *h_v_* of 0.08 m was used to simulate open channel vegetation. It should be noted that this study focuses on the influence of the combined and discrete distribution of stem of patch vegetation on the channel flow structure, and the factors related to vegetation flexibility are not included in the numerical model of this study. Instead, the simulated vegetation is set as rigid and non-deformable. However, since vegetation is usually distributed vertically at the bottom of the channel, its unique morphological distribution has a different impact on the water flow than the stone particles at the bottom of the channel. The influence on the water flow structure of the open channel partly comes from the distribution characteristics of stone particles at the bottom of the channel, and the other part comes from the morphological characteristics of the vegetation. The unique vertical morphological characteristics of vegetation is the main factor causing the significant change of vertical flow structure of open channel, that is, the presence of vegetation changes the mixing and energy dissipation effect of open channel flow. This study considered the vegetation layout of two different combinations of stem thickness under different states, non-submerged and submerged (**Figure 1**). The x, y, and z axes represent the longitudinal, transverse, and vertical directions, respectively. The vegetation layout in the numerical calculation area is shown in **Figure 2**.

The calculation model adopts the boundary conditions of the velocity inlet (0.3 m·s^-1^) and the pressure outlet, the upper surface uses the slip edge to replace the water-air interface, and the vegetation surface and the wall both use the standard wall function, which was a non-slip boundary. Simulation and post-processing were performed in FLUENT. The pressure-velocity coupling was determined using the SIMPLE algorithm, whereby the relaxation factors of kinetic energy and pressure were 0.7 and 0.3 respectively, the flow velocity was 0.7, the Reynolds stress was 0.5, and the turbulent kinetic energy and turbulent dissipation rate were both 0.8. The minimum residual value of each equation was set to 1×10^-5^, that is, the iteration ended when the calculated residual value was less than the minimum residual value. Therefore, using the above criteria, it was assumed that the solution had reached a steady state.

**Figure 3:**
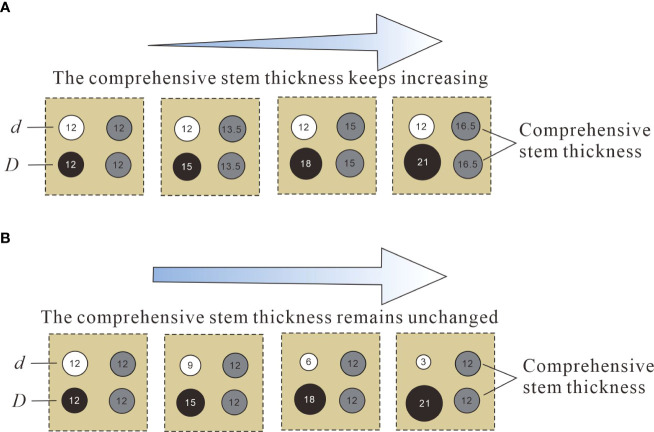
Creative diagram of the distributions of two different vegetation combinations. **(A)** Vegetation combination distribution, **(B)** Vegetation discrete distribution.

In order to further study the effect of the combined vegetation comprehensive stem thickness change on the flow characteristics of the open channel, the model was setup with four vegetation combination distributions with different comprehensive stem thicknesses (*d* & *D)* which were 0.012 m & 0.012 m, 0.012 m & 0.015 m, 0.012 m & 0.018 m, and 0.012 m & 0.021 m. Under the premise that the comprehensive stem thickness of vegetation remains unchanged, the discrete degree of stem thickness of adjacent plants will inevitably become an inducing factor for the change of water flow characteristics. To study the influence of vegetation stem dispersion degree on the flow characteristics of an open channel, the model was set up with four different discrete stem diameter distributions, 0.012 m & 0.012 m, 0.009 m & 0.015 m, 0.006 m & 0.018 m, and 0.003 m & 0.021 m. In this study, both vegetation combination and discrete distribution belong to two types of combined distribution of different stem diameter vegetation, but there are essential differences between them from the perspective of research emphasis. The combined distribution study is based on the premise that the comprehensive stem thickness is constantly changing to explore the effect of the vegetation comprehensive stem thickness on the flow characteristics of the open channel, the so-called comprehensive stem thickness is the average diameter of two kinds of combined vegetation (**Figure 3A**). The discrete distribution study is based on the premise that the comprehensive stalk thickness of the vegetation is constant, to explore the influence of the degree of stem thickness differentiation between the two types of vegetation on the water flow characteristics of the open channel (**Figure 3B**).

### 2.2 Validation of numerical simulation methods

Based on the open channel flume experiment, a hydraulic model was established indoors. The length (*x*-axis), width (*y*-axis), and height (*z*-axis) of the open channel flume were 5m, 0.4m, and 0.3m, respectively, and the experimental slope was 0.0%.The flow discharges under vegetation non-submerged and submerged conditions were 0.0072 m^3^·s^-1^ and 0.012 m^3^·s^-1^, respectively. By adjusting the tailgate at the downstream end of the flume, the water depth in the flume was adjusted so that the water depth at 1.64 m of the flume length was consistent with that at the corresponding inlet of the model, so that the inlet velocity of the model should be 0.3 m·s^-1^. In order to homogenize the flow discharge, measurements were made every ten minutes in the experiment. Cover the entire bottom of the flume with a PVC baseboard. Rigid cylinders (diameter *d* & *D* is 0.012 m & 0.012 m, 0.012 m & 0.015m, 0.012m & 0.018m, 0.012m & 0.021 m, 0.009 m & 0.015 m, 0.006 m & 0.018 m, 0.003 m & 0.021 m) were used to simulate rigid vegetation and were intermittently fixed on the experimental floor in the form of patches. Additionally, 3D acoustic doppler velocimeter (3D ADV) is used to measure the velocity at specific positions P_1_ and P_2_ in **Figure 2**.

**Figure 4:**
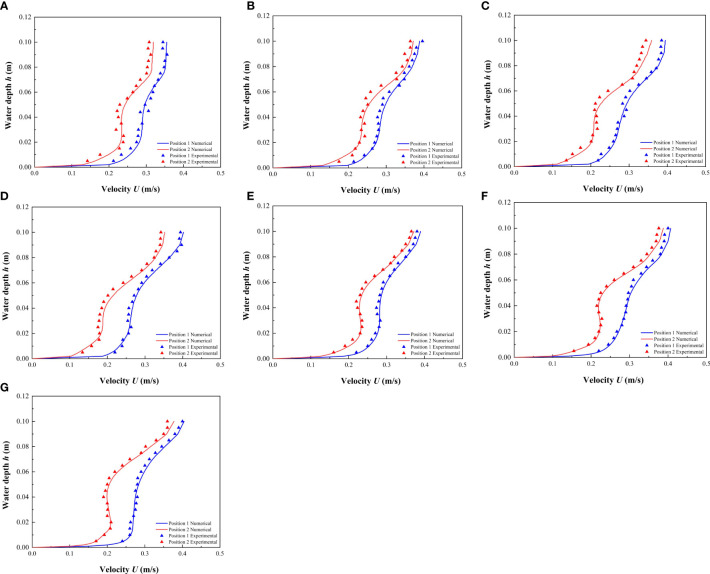
Comparison between the experimental results and the numerical results of the modeling method used in this study. **(A)** 0.012 m & 0.012 m, **(B)** 0.012 m & 0.015 m, **(C)** 0.012 m & 0.018 m, **(D)** 0.012 m & 0.021 m, **(E)** 0.009 m & 0.015 m, **(F)** 0.006 m & 0.018 m, **(G)** 0.003 m & 0.021 m.

Under the conditions of different combinations and discrete distribution of vegetation, the average velocity calculation results obtained at the specified location were compared with the experimental results (**Figure 4**). The results showed that the experimental and simulated data were similar, which verifies the accuracy of the numerical simulation method, and further revealed that the numerical model could simulate the flow of open channels with discontinuous vegetation patches.

## 3 Results and discussion

### 3.1 Longitudinal distribution of streamwise velocity before and after a single vegetation

**Figure 5:**
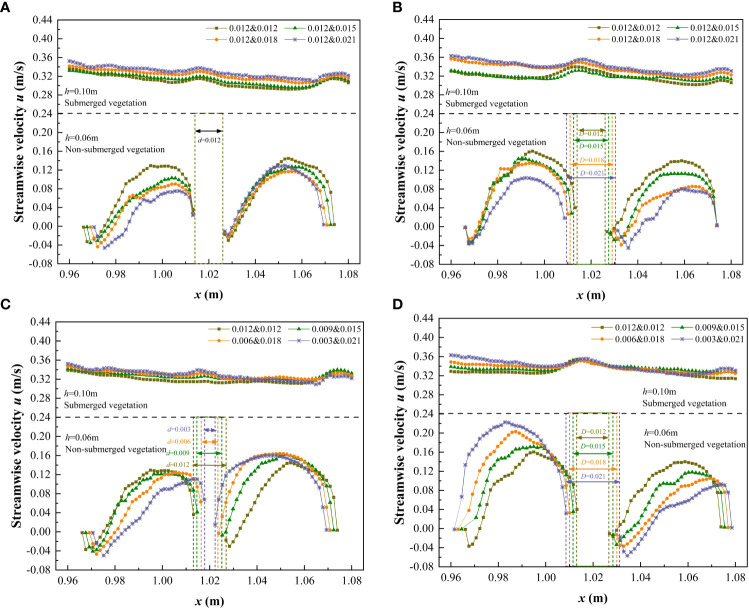
Variation streamwise velocity before and after a single vegetation (*h* = 0.06 m and 0.10 m) under different submerged conditions. **(A)** L_1_, vegetation combination distribution, **(B)** L_2_, vegetation combination distribution, **(C)** L_1_, vegetation discrete distribution, **(D)** L_2_, vegetation discrete distribution.

**Figure 5** shows the longitudinal distribution of the streamwise velocity before and after a single vegetation in patches with *h* of 0.06m (non-submerged condition) and 0.10m (submerged condition), that is, at the L_1_ and L_2_ positions, the lengths of L_1_ and L_2_ were 0.12 m. For this study, the central position of L_1_ represented the smaller diameter plant *d* (the combined vegetation was 0.012 m, and the discrete vegetation sizes were 0.012 m, 0.009 m, 0.006 m, and 0.003 m), and the central position of L_2_ represented the larger diameter plant *D* (the combined vegetation corresponds to 0.012 m, 0.015 m, 0.018 m, and 0.021 m, and the discrete vegetation corresponds to 0.012 m, 0.015 m, 0.018 m, 0.021 m). This setup was used to study the influence of combination and discrete characteristics of longitudinal discontinuous patches on streamwise velocity.

It can be seen from **Figure 5** that under non-submerged conditions (*h*<*h_v_*), upstream streamwise velocity decreased when approaching the front edge of vegetation, and the streamwise velocity rapidly decreased to a minimum value behind (trailing edge) the vegetation, and then gradually increased until the water reached the next plant for velocity circulation. The flow velocity shows the typical characteristics of the wake downstream of the cylindrical obstacle, and the distance from the trailing edge of the vegetation to the longitudinal position where the streamwise velocity decreased to a minimum value represents the length of the stable wake zone. The formation of the stable wake zone is primarily due to the shear layer originating from the shoulders on both sides of the cylinder, which isolates the high-speed water flowing around from both sides of the cylinder outside the wake zone, therefore the momentum in the stable wake zone was relatively low. When the shear layers on both sides are wide enough to intersect, the wake flow velocity begins to recover and the stable wake zone ends, that is, the end point of the stable wake zone corresponds to the position where the shear layers intersect. It can be concluded from **Figure 5** that the length of the stable wake zone is closely related to the stem thickness of vegetation. For the combined vegetation patches, when the stem diameter of vegetation remained unchanged at 0.012 m, the length of the stable wake zone was unchanged at approximately 0.0015 m (**Figure 5A**). When the stem diameter of vegetation increased from 0.012 m to 0.021 m, the length of the stable wake zone also increased from 0.0015 m to 0.0030 m (**Figures 5B, D**). When the stem diameter of vegetation was < 0.012 m, the length of the wake zone at the trailing edge of vegetation was very small (**Figure 5C**). Therefore, we concluded that the diameter of vegetation is an important factor affecting the wake structure.The research results are of great significance for understanding the flow structure around the flow field in the vegetation unit area.

Under the non-submerged condition, for the distribution of four different combinations of vegetation in the patch, the difference in streamwise velocity behind the vegetation was low when the stem thickness of vegetation was constant. Further, the streamwise velocity behind the vegetation decreased as the stem thickness of vegetation increased. Regarding the distribution of the four different discrete vegetation types in the patch, the streamwise velocity behind the vegetation was negatively correlated with the thickness of the vegetation stem, that is, the larger the stem thickness, the smaller the streamwise velocity. Additionally, as the vegetation dispersion increased so did the difference in streamwise velocity front and behind the vegetation, indicating that the discrete distribution of vegetation somewhat interferes with the longitudinal continuity of the velocity, and destroys the uniformity of velocity in the longitudinal flow field.

Under the complete submersion (*h*>*h_v_*) condition, the higher the comprehensive stem thickness of the combined vegetation, the higher the streamwise velocity corresponding to the free layer, that is, *u*_0.012&0.012_<*u*_0.012&0.015_<*u*_0.012&0.018_<*u*_0.012&0.021_. For the discrete distribution of vegetation, the greater the degree of vegetation dispersion, the greater the streamwise velocity corresponding to the free layer, that is, *u*_0.012&0.012_<*u*_0.009&0.015_<*u*_0.006&0.018_<*u*_0.003&0.021_. This occurred because as the comprehensive stem thickness and degree of vegetation dispersion increases, the density of vegetation coverage also increases, which hinders the water flow in the vegetation layer and produces a strong lateral divergent flow in the free layer leading to an elevated streamwise velocity in the free layer. Of note, in the submerged state, because the free layer is less affected by the water blocking effect of vegetation, the difference in streamwise velocity under different combinations and discrete forms is not clear, which further indicates that in the submerged state, the influence of vegetation distribution patterns on the flow characteristics of the free layer is weakened. It also shows that the influence of vegetation heterogeneity on the difference of water flow characteristics is closely related to the submerged state.

### 3.2 Velocity distribution in specific cross-sections

#### 3.2.1 Streamwise velocity distribution in specific cross-sections

**Figure 6:**
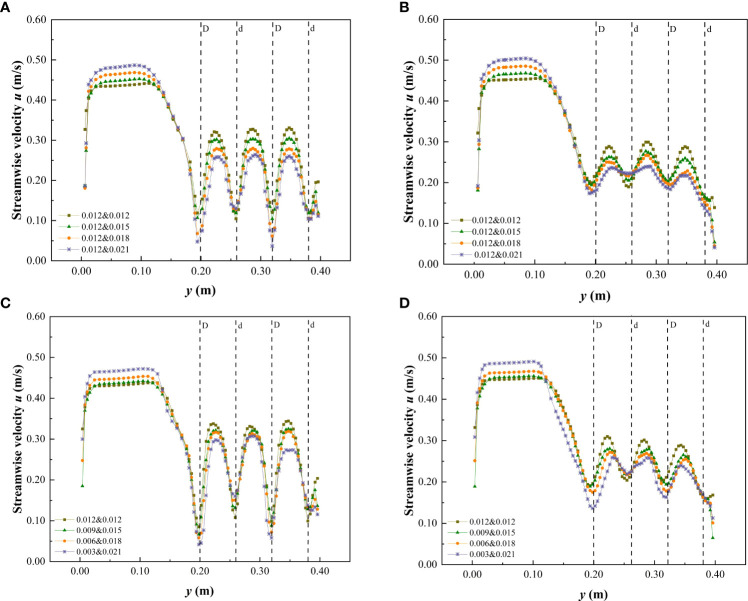
Variation of streamwise velocity in cross-section when *h* is 0.06 m under the non-submerged condition. **(A)** C_2_, vegetation combination distribution, **(B)** C_3_, vegetation combination distribution, **(C)** C_2_, vegetation discrete distribution, **(D)** C_3_, vegetation discrete distribution.

**Figure 7:**
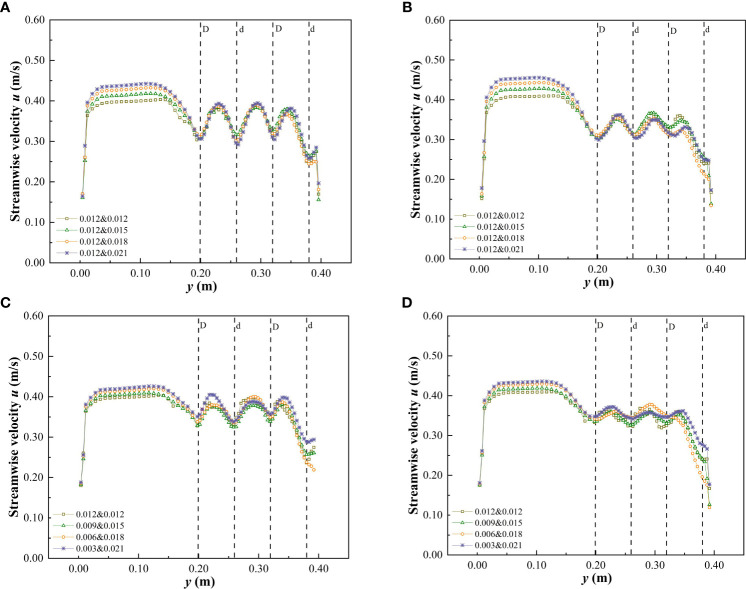
Variation of streamwise velocity in cross-section when *h* is 0.10 m under the submerged condition. **(A)** C_2_, vegetation combination distribution, **(B)** C_3_, vegetation combination distribution, **(C)** C_2_, vegetation discrete distribution, **(D)** C_3_, vegetation discrete distribution.

The cross-sections C_2_ and C_3_ at the middle of the third patch area and the middle of the spaced area behind the patch, respectively, were selected to measure the change of flow velocity distribution. The streamwise velocity of a specific water depth on the cross-section under different submerged conditions is shown in **Figures 6**, **7**. Under the non-submerged vegetation condition, the streamwise velocity in the non-vegetated area was significantly higher than that in the vegetated area (**Figure 6**). **Table 1** also shows that the average streamwise velocity in the non-vegetated area was significantly higher than that in the vegetated area, indicating a strong energy exchange between vegetated and non-vegetated areas in an open channel with patchy distributions of partially discontinuous combined vegetation, similar to the findings of Huai et al. (2019) and Tang et al. (2021). In addition, when the water flow encountered cylindrical obstacles, there was strong lateral divergence with high streamwise velocity at narrow channels between the vegetation (**Figures 6A, C**). When water flow was not blocked by cylindrical obstacles, the amplitude of streamwise velocity in the C_3_ spaced area decreased more than when compared with that in the C_2_ patch area, but it was still uneven (**Figures 6B, D**), indicating that the patch influence still existed in a certain distance beyond the downstream edge.

**Table 1:** The average streamwise velocity $\bar{u}$ value and its rate of change between C_1_ and C_2_ cross-sections under different distribution patterns when the non-submerged condition *h* is 0.06 m.

	Vegetation non-submerged state (*h*=0.06m)				
**C_2_**	**Combined distribution (*d*&*D*)**	${\bar{u}}_{vegetation\;area}$	**Growth rate of ${\bar{u}}_{vegetation\;area}$ relative 0.012&0.012 (%)**	${\bar{u}}_{non-vegetated\;area}$	${\bar{u}}_{non-vegetated\;area}$ **/** ${\bar{u}}_{vegetated\;area}$
	0.012&0.012	0.2415	--	0.4371	1.8102
	0.012&0.015	0.2243	-7.12%	0.4468	1.9924
	0.012&0.018	0.2050	-15.08%	0.4623	2.2544
	0.012&0.021	0.1854	-23.20%	0.4792	2.5843
**C_3_**	**Combined distribution (*d*&*D*)**	${\bar{u}}_{vegetation\;area}$	**Growth rate of ${\bar{u}}_{vegetation\;area}$ relative 0.012&0.012 (%)**	${\bar{u}}_{non-vegetated\;area}$	${\bar{u}}_{non-vegetated\;area}$ **/** ${\bar{u}}_{vegetated\;area}$
	0.012&0.012	0.2368	--	0.4525	1.9104
	0.012&0.015	0.2254	-4.84%	0.4627	2.0530
	0.012&0.018	0.2132	-9.97%	0.4791	2.2468
	0.012&0.021	0.2010	-15.13%	0.4969	2.4723
**C_2_**	**Discrete distribution (*d*&*D*)**	${\bar{u}}_{vegetation\;area}$	**Growth rate of ${\bar{u}}_{vegetation\;area}$ relative 0.012&0.012 (%)**	${\bar{u}}_{non-vegetated\;area}$	${\bar{u}}_{non-vegetated\;area}$ **/** ${\bar{u}}_{vegetated\;area}$
	0.012&0.012	0.2491	--	0.4336	1.7409
	0.009&0.015	0.2362	-5.15%	0.4376	1.8524
	0.006&0.018	0.2295	-7.85%	0.4492	1.9574
	0.003&0.021	0.2136	-14.25%	0.4682	2.1924
**C_3_**	**Discrete distribution (*d*&*D*)**	${\bar{u}}_{vegetation\;area}$	**Growth rate of ${\bar{u}}_{vegetation\;area}$ relative 0.012&0.012 (%)**	${\bar{u}}_{non-vegetated\;area}$	${\bar{u}}_{non-vegetated\;area}$ **/** ${\bar{u}}_{vegetated\;area}$
	0.012&0.012	0.2419	--	0.4487	1.8550
	0.009&0.015	0.2296	-5.10%	0.4520	1.9693
	0.006&0.018	0.2219	-8.27%	0.4646	2.0939
	0.003&0.021	0.2096	-13.35%	0.4872	2.3243

In the non-submerged state, the combination and discrete distribution of patch vegetation were the important factors affecting the change of streamwise velocity in the cross-section. The streamwise velocity at C_2_ and C_3_ in the vegetated area showed the same change rule, that is, the streamwise velocity at the vegetated channel in the patch area decreased continuously as the comprehensive stem thickness of the combined vegetation and the degree of vegetation dispersion increased. It can also be concluded from **Figures 6A, C** that the flow velocity behind the large-diameter vegetation in the non-submerged state was lower than that behind the small-diameter vegetation (at C_2_). This demonstrates that the trailing edge of thicker vegetation is more conducive to the accumulation of sediment and provides a good environment for vegetation growth (Shi et al., 2016). In addition, the streamwise velocity in the non-vegetated area was also somewhat affected by the vegetation distribution form in the vegetated area, that is, with the increase of the comprehensive stem thickness and discrete degree of the combined vegetation, the streamwise velocity in the non-vegetated area showed an increasing trend. **Table 1** shows that as the comprehensive stem thickness of the combined vegetation and the degree of dispersion increase, the average streamwise velocity of the cross-sections C_2_ and C_3_ in the vegetated area decreased, while the average streamwise velocity in the non-vegetated area showed an increasing trend.

When compared with the non-submerged state, the difference in flow velocity between the non-vegetated and vegetated areas was reduced in the submerged state (**Figure 7** and **Table 2**), indicating that the energy exchange between the two areas was reduced in the submerged state. In addition, the combination and discrete vegetation distributions showed no clear effect on the streamwise velocity differences in the vegetated area, while in the non-vegetated area the streamwise velocity retained the variation law that it would increase as the comprehensive stem thickness and discrete degree of the combined vegetation increased.

**Table 2:** The average streamwise velocity $\bar{u}$ value and its rate of change between C_1_ and C_2_ cross-sections under different distribution patterns when the submerged condition *h* is 0.10 m.

	Vegetation submerged state (*h*=0.10m)				
**C_2_**	**Combined distribution (*d*&*D*)**	${\bar{u}}_{vegetation\;area}$	**Growth rate of ${\bar{u}}_{vegetation\;area}$ relative 0.012&0.012 (%)**	${\bar{u}}_{non-vegetated\;area}$	${\bar{u}}_{non-vegetated\;area}$ **/** ${\bar{u}}_{vegetated\;area}$
	0.012&0.012	0.3356	--	0.3978	1.1852
	0.012&0.015	0.3407	1.52%	0.4138	1.2145
	0.012&0.018	0.3349	-2.24%	0.4278	1.2775
	0.012&0.021	0.3396	11.93%	0.4381	1.2899
**C_3_**	**Combined distribution (*d*&*D*)**	${\bar{u}}_{vegetation\;area}$	**Growth rate of ${\bar{u}}_{vegetation\;area}$ relative 0.012&0.012 (%)**	${\bar{u}}_{non-vegetated\;area}$	${\bar{u}}_{non-vegetated\;area}$ **/** ${\bar{u}}_{vegetated\;area}$
	0.012&0.012	0.3201	--	0.4073	1.2723
	0.012&0.015	0.3221	0.62%	0.4255	1.3210
	0.012&0.018	0.3084	-3.67%	0.4404	1.4283
	0.012&0.021	0.3162	-1.22%	0.4526	1.4314
**C_2_**	**Discrete distribution (*d*&*D*)**	${\bar{u}}_{vegetation\;area}$	**Growth rate of ${\bar{u}}_{vegetation\;area}$ relative 0.012&0.012 (%)**	${\bar{u}}_{non-vegetated\;area}$	${\bar{u}}_{non-vegetated\;area}$ **/** ${\bar{u}}_{vegetated\;area}$
	0.012&0.012	0.3482	--	0.3978	1.1425
	0.009&0.015	0.3408	-2.11%	0.4053	1.1892
	0.006&0.018	0.3450	-0.90%	0.4145	1.2012
	0.003&0.021	0.3607	3.59%	0.4208	1.1667
**C_3_**	**Discrete distribution (*d*&*D*)**	${\bar{u}}_{vegetation\;area}$	**Growth rate of ${\bar{u}}_{vegetation\;area}$ relative 0.012&0.012 (%)**	${\bar{u}}_{non-vegetated\;area}$	${\bar{u}}_{non-vegetated\;area}$ **/** ${\bar{u}}_{vegetated\;area}$
	0.012&0.012	0.3272	--	0.4073	1.2450
	0.009&0.015	0.3239	-14.62%	0.4162	1.2847
	0.006&0.018	0.3239	-21.04%	0.4267	1.3172
	0.003&0.021	0.3410	6.84%	0.4330	1.2697

This study can understand the overall change of the flow field velocity in the vegetated area and the non-vegetation area under different combinations of vegetation, and provide a certain scientific basis for the research on the mechanism of riverbank sediment erosion and deposition and the growth and evolution trend of vegetation communities, which can be applied to the restoration and management of actual river channels.

#### 3.2.2 Lateral velocity distribution in specific cross-sections

**Figure 8:**
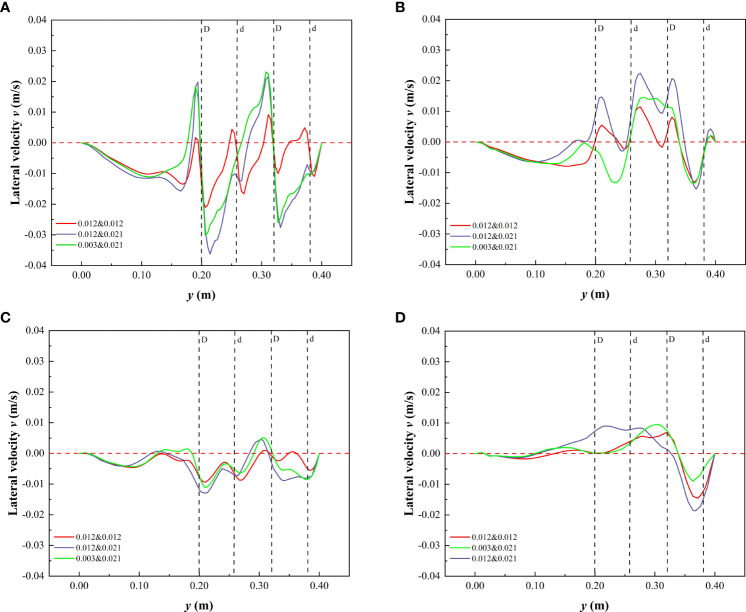
Variation of lateral velocity in cross-section at h of 0.06 m and 0.10 m under different submerged conditions. **(A)** C_2_, *h* = 0.06 m, non-submerged vegetation, **(B)** C_2_, *h* = 0.10 m, submerged vegetation, **(C)** C_3_, *h* = 0.06 m, non-submerged vegetation, **(D)** C_3_, *h* = 0.10 m, submerged vegetation.

For open channels with partial discontinuous combined vegetation patches, the unique distribution was expected to have a different impact on the lateral velocity of water flow. **Figure 8** shows the variation in lateral velocity of cross-sections C_2_ and C_3_ in specific water depths under different submerged conditions. The lateral velocity at C_2_ under the vegetated non-submerged state is shown in **Figure 8A**. When the vegetation stem thickness was uniform, the lateral velocity variation in the patch area was low. As the comprehensive vegetation stem thickness and the discrete degree of the vegetation stem increased, the lateral velocity variation in the patch area also increased, while the vegetation combination and discrete distribution form at C_3_ of the spaced area had minimal effect on the transverse velocity of the y-section (**Figure 8C**). In addition, in the non-submerged state, the lateral flow velocity of the y-section was primarily negative (**Figures 8A, C**), indicating that the direction of the overall lateral velocity was towards the non-vegetated area. In the submerged state, the lateral velocity of the free layer was minimally affected by the vegetation combination and discrete distribution form of the vegetation layer, and the lateral velocity was predominantly positive (**Figures 8B, D**), indicating that the direction of the overall lateral velocity was towards the side of the vegetated area. The distribution of vegetation along the riverbank is a common feature of natural rivers and artificial ecological rivers. The study of lateral flow velocity in the flow field is helpful to understand the lateral transport of sediment and to improve the navigation of the river.

### 3.3 Spatial distribution of velocity contours in different sections

**Figure 9:**
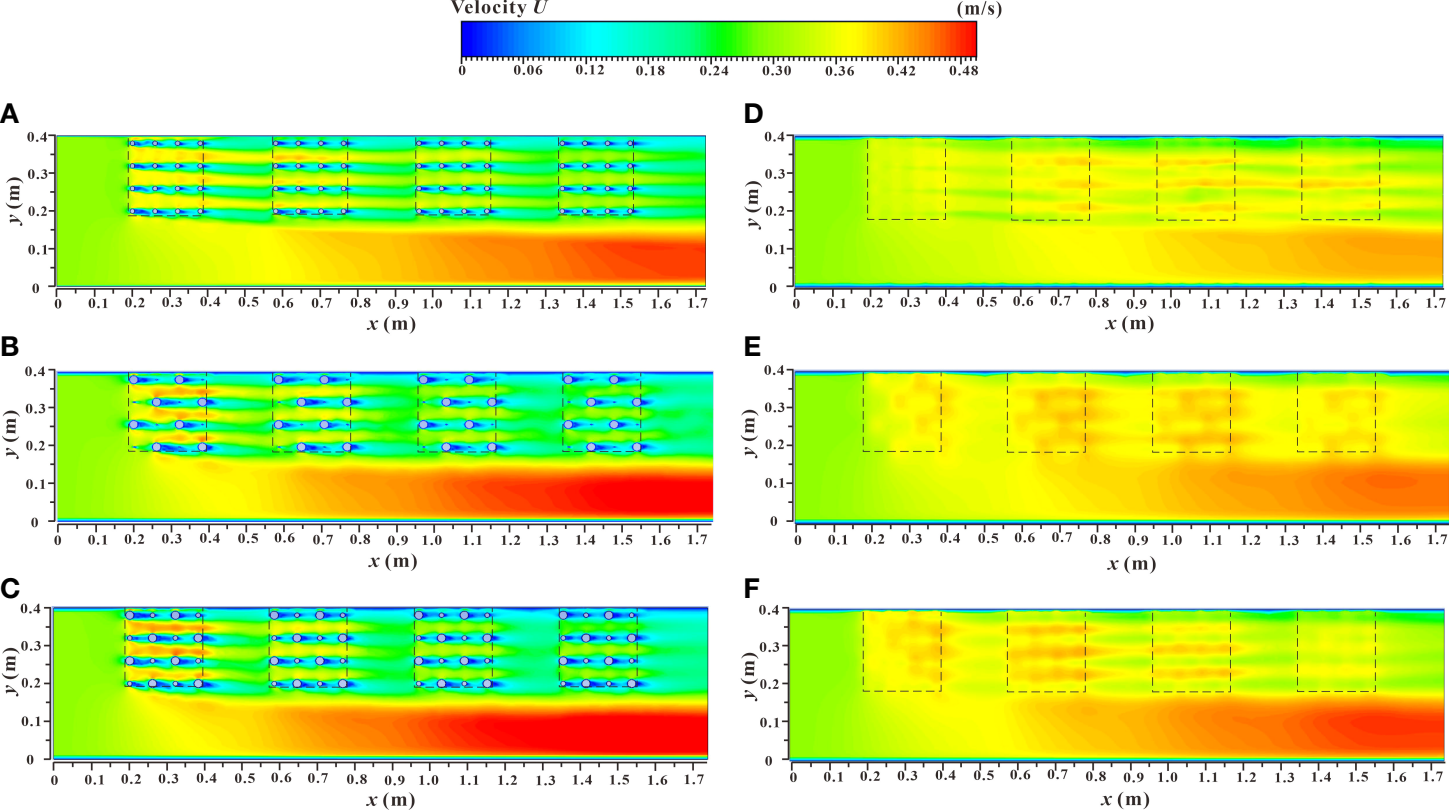
Spatial distribution of velocity contours of xy sections under different submerged states. **(A)** *h* = 0.06 m, d & *D* = 0.012 m & 0.012 m, non-submerged vegetation, **(B)** *h* = 0.06 m, d & *D* = 0.003 m & 0.021 m, non-submerged vegetation, **(C)** *h* = 0.06 m, d & *D* = 0.012 m & 0.021 m, non-submerged vegetation, **(D)** *h* = 0.10 m, d & *D* = 0.012 m & 0.012 m, submerged vegetation, **(E)** *h* = 0.10 m, d & *D* = 0.003 m & 0.021 m, submerged vegetation, **(F)** *h* = 0.10 m, d & *D* = 0.012 m & 0.021 m, submerged vegetation.

**Figure 10:**
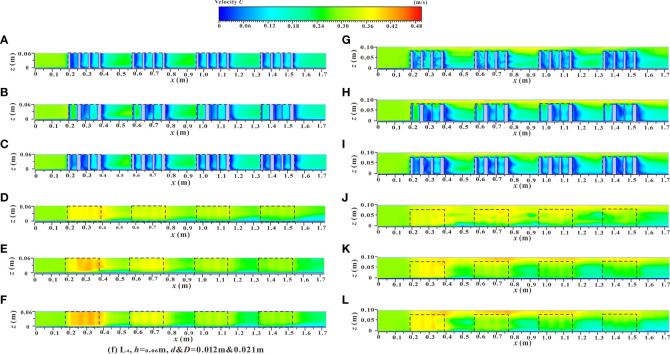
Spatial distribution of velocity contours of yz sections under different submerged states. **(A)** L_3_, *h* = 0.06 m, d & *D* = 0.012 m & 0.012 m, non-submerged vegetation, **(B)** L_3_, *h* = 0.06 m, d & *D* = 0.003 m & 0.021 m, non-submerged vegetation, **(C)** L_3_, *h* = 0.06 m, d & *D* = 0.012 m & 0.021 m, non-submerged vegetation, **(D)** L_4_, *h*= 0.06 m, d & *D* = 0.012 m & 0.012 m, non-submerged vegetation, **(E)** L_4_, *h* = 0.06 m, d & *D* = 0.003 m & 0.021 m, non-submerged vegetation, **(F)** L_4_, *h* = 0.06 m, d & *D* = 0.012 m & 0.021 m, non-submerged vegetation, **(G)** L_3_, *h* = 0.10 m, d & *D* = 0.012 m & 0.012 m, submerged vegetation, **(H)** L_3_, *h* = 0.10 m, d & *D* = 0.003 m & 0.021 m, submerged vegetation, **(I)** L_3_, *h* = 0.10 m, d & *D* = 0.012 m & 0.021 m, submerged vegetation, **(J)** L_4_, *h* = 0.10 m, d & *D* = 0.012 m & 0.012 m, submerged vegetation, **(K)** L_4_, *h* = 0.10 m, d & *D* = 0.003 m & 0.021 m, submerged vegetation, **(L)** L_4_, h = 0.10 m, d & *D* = 0.012 m & 0.021 m, submerged vegetation.

The spatial distribution of the velocity contours of the xy and yz profiles (C_1_ and C_2_) under two different submerged states are shown in **Figures 9**, **10**. From the figures, it can be deduced that the submerged state and distribution pattern of vegetation are important factors that affect the velocity change and energy exchange of the two water passages in the vegetated and the non-vegetated areas. In the non-submerged state, the velocity in the vegetated area was significantly lower than that in the non-vegetated area and the velocity difference was high. In the submerged state, the velocity difference was significantly reduced, indicating that there was a strong energy exchange at the interface between the vegetated and the non-vegetated areas, while in the submerged state, the energy exchange was weakened. In addition, the increases in the comprehensive stem thickness of the combined vegetation and the dispersion degree were also important factors causing the increased velocity difference between the vegetated and the non-vegetated areas. **Figure 9** also reflects the difference in flow velocity along the xy profile. In the non-submerged state, the overall flow velocity in the vegetated area gradually decreased as the downstream distance increased, while the overall flow velocity in the non-vegetated area showed the opposite trend. Under the submerged state, the overall flow velocity in the non-vegetated area was similar to that of the non-submerged state, while the overall flow velocity in the vegetated area had no clear regularity.

**Figure 11:**
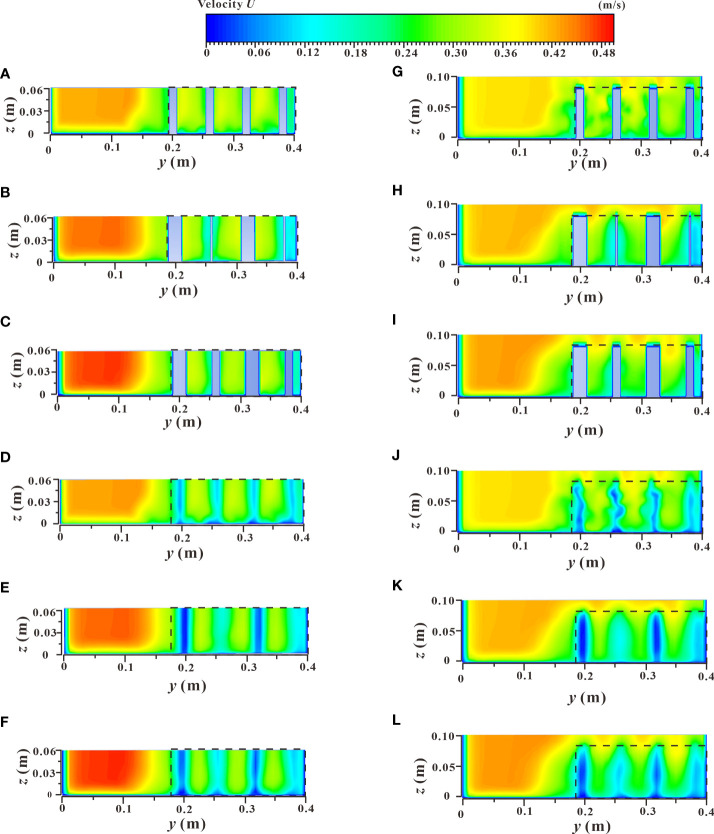
Spatial distribution of velocity contours of xz sections under different submerged states. **(A)** C_1_, *h* = 0.06 m, d & *D* = 0.012 m & 0.012 m, non-submerged vegetation, **(B)** C_1_, *h* = 0.06 m, d & *D* = 0.003 m & 0.021 m, non-submerged vegetation, **(C)** C_1_, *h* = 0.06 m, d & *D* = 0.012 m & 0.021 m, non-submerged vegetation, **(D)** C_2_, *h* = 0.06 m, d & *D* = 0.012 m & 0.012 m, non-submerged vegetation, **(E)** C_2_, *h* = 0.06 m, d & *D* = 0.003 m & 0.021 m, non-submerged vegetation, **(F)** C_2_, *h* = 0.06 m, d & *D* = 0.012 m & 0.021 m, non-submerged vegetation, **(G)** C_1_, *h* = 0.10 m, d & *D* = 0.012 m & 0.012 m, submerged vegetation, **(H)** C_1_, *h* = 0.10 m, d & *D* = 0.003 m & 0.021 m, submerged vegetation, **(I)** C_1_, *h* = 0.10 m, d & *D* = 0.012 m & 0.021 m, submerged vegetation, **(J)** C_2_, *h* = 0.10 m, d & *D* = 0.012 m & 0.012 m, submerged vegetation, **(K)** C_2_, *h* = 0.10 m, d & *D* = 0.003 m & 0.021 m, submerged vegetation, **(L)** C_2_, *h* = 0.10 m, d & *D* = 0.012 m & 0.021 m, submerged vegetation.

The spatial distribution of the velocity contours of the xz profile under two different submerged states is shown in **Figure 11**, which shows the velocity distribution changes along the longitudinal profile of the vegetation array (L_3_), and the longitudinal profile of the vegetation passage (L_4_) under different vegetation distribution modes. In the non-submerged state, when water flowed through the vegetation array (L_3_), the influence of the wake area behind the cylinder caused the velocity to decline to a minimum value. This resulted in a significantly lower flow velocity in the vegetation patch than in the spaced area between adjacent patches (**Figures 11A–C**), which led to sediment deposition and increased availability of soil nutrients and ultimately a suitable environment for the growth and reproduction of vegetation.

When the water flow passed through the vegetation passage (L_4_), the influence of vegetation shear force caused a narrowing of the passage which resulted in a higher flow velocity in the vegetation patch than that in the spaced area (**Figures 11D–F**). From an ecological perspective, the low flow velocity area was more suitable for the survival of aquatic organisms (Widdows & Navarro, 2007; Folkard & Gascoigne, 2009), concluded from the **Figures 11A–F**, in the process of water flow downstream, the velocity of the vegetation array (L_3_) and the vegetation passage (L_4_) in the patch area and the spaced area showed a trend of decreasing gradually with the increase of the downstream distance, and the flow velocity was more moderate in the downstream parts of the river. In terms of the vegetation distribution of partial discontinuous patches in the river, the velocity distribution areas of specific patches and the rear spaced area from high to low were as follows: L_4_ patch area > L_4_ spaced area > L_3_ spaced area > L_3_ patch area. In addition, the increased comprehensive stalk thickness and discrete degree of the combined vegetation resulted in a greater obstruction to water flow by the vegetation. This led to a stronger sedimentation effect and increased the water carrying capacity of the river channel. The velocity distribution of the xz longitudinal profile under the submerged condition (**Figures 11G–L**) was consistent with that under the non-submerged condition. As shown in **Figures 11G–L** and **Figures 10G–L**, there was a significant difference in flow velocity between the upper and lower layers (vegetation layer and free layer), bounded by the top of the vegetation in the submerged state, indicating a strong mixed layer near the top of the vegetation and a strong energy exchange occurs.

### 3.4 Turbulence characteristics

#### 3.4.1 Vertical distribution of Reynolds stress at specific locations of vegetation patch channels

Reynolds stress is the additional shear stress caused by the mixing of turbulent liquid particles, which is closely related to the flow structure and distribution of vegetation in the river channel (Liu et al., 2010; Li et al., 2020; Caroppi et al., 2022). Reynolds stress can also be caused by the uneven distribution of the flow field in space and is the statistical average value of the momentum per unit time and unit area caused by turbulent flow pulsation (Afzalimehr et al., 2010). Therefore, Reynolds stress is an indispensable factor in the study of the characteristics of channel flow.

**Figure 12:**
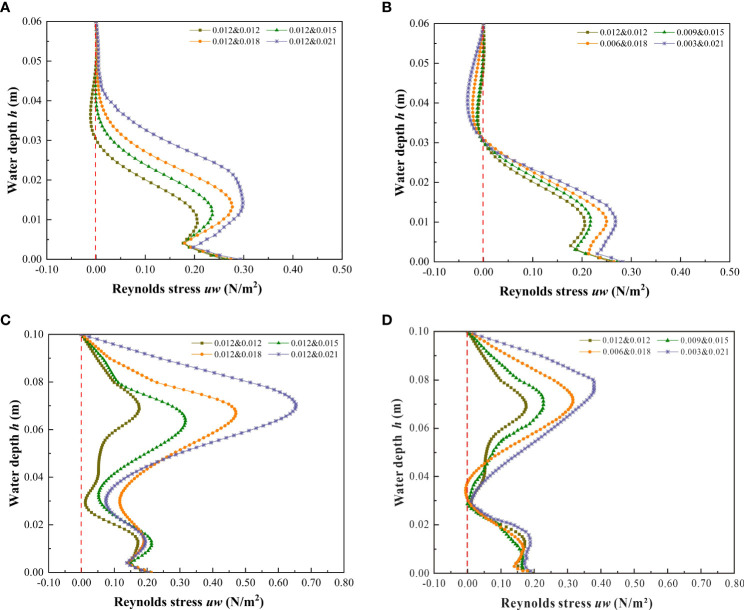
Vertical distribution of Reynolds stress at the location of vegetation patch channel P_1_ under different submerged states. **(A)** *h* = 0.06 m, vegetation combination distribution, non-submerged vegetation, **(B)** *h* = 0.06 m, vegetation discrete distribution, non-submerged vegetation, **(C)** *h* = 0.10 m, vegetation combination distribution, submerged vegetation, **(D)** *h* = 0.10 m, vegetation discrete distribution, submerged vegetation.

**Figure 12** shows the vertical distribution of Reynolds stress at the vegetation patch channel P_1_ under different submerged states. For the unsubmerged vegetation (**Figures 12A, B**), the Reynolds stress is higher near the bottom of the channel and the bottom of the vegetation where *h* is approximately 0.015 m (1/4 of the overall water depth), and further decreases at the bottom of the channel (*h* is from 0 to 0.005 m) due to the large resistive shear force caused by the riverbed (Dey, 2014). When *h* > 0.005 m, the Reynolds stress first increases then decreases, reaching the lowest point when *h* is approximately 0.015 m. For the vegetation combination distribution (**Figure 12A**), when *h* was 0.015 m, *d*&*D* were 0.012 m & 0.015 m, 0.012 m & 0.018 m, and 0.012 m & 0.021 m, and the Reynolds stress growth rate was 14.90%, 34.61%, and 44.92%, respectively, compared to 0.012 m & 0.012 m (**Table 3**). When the water depth is approximately 0.05 m to the water surface, the vegetation combination distribution has almost no effect on the change of Reynolds stress, which is effectively 0. For the discrete distribution of vegetation (**Figure 12B**), *h* is at 0.015 m, *d*&*D* is 0.009 m & 0.015 m, 0.006 m & 0.018 m, and 0.003 m & 0.021 m, and the Reynolds stress growth rate is 5.60%, 21.45%, and 30.28%, respectively, compared to 0.012 m & 0.012 m (**Table 3**), when the water depth is approximately 0.03 m to the water surface, the discrete vegetation distribution has almost no effect on the change of Reynolds stress, which is effectively 0.

**Table 3:** The Reynolds stress *uw* value and its rate of change in specific water depths at P_1_ position under different submerging states and distribution modes.

Vegetation non-submerged state (*h*=0.06m)					
**Combined distribution *d*&*D* (m)**	***uw* (*h*=0.015m)**	**Growth rate of *uw* relative 0.012&0.012 (%)**	**Discrete distribution *d*&*D* (m)**	***uw* (*h*=0.015m)**	**Growth rate of *uw* relative 0.012&0.012 (%)**
0.012&0.012	0.2057	--	0.012&0.012	0.2057	--
0.012&0.015	0.2364	14.90%	0.009&0.015	0.2173	5.60%
0.012&0.018	0.2770	34.61%	0.006&0.018	0.2499	21.45%
0.012&0.021	0.2982	44.92%	0.003&0.021	0.2680	30.28%
**Vegetation submerged state (*h*=0.10m)**					
**Combined distribution *d*&*D* (m)**	***uw* (*h*=0.015m)**	**Growth rate of *uw* relative 0.012&0.012 (%)**	**Discrete distribution *d*&*D* (m)**	***uw* (*h*=0.015m)**	**Growth rate of *uw* relative 0.012&0.012 (%)**
0.012&0.012	0.1759	--	0.012&0.012	0.1758	--
0.012&0.015	0.3169	80.13%	0.009&0.015	0.2280	29.67%
0.012&0.018	0.4705	167.41%	0.006&0.018	0.3161	79.79%
0.012&0.021	0.6537	271.56%	0.003&0.021	0.3795	115.87%

The submerged state of vegetation is also an important factor affecting Reynolds stress distribution. For submerged vegetation (**Figures 12C, D**), the Reynolds stress reaches a maximum value near the top of the vegetation canopy, indicating that the shear stress inside the water flow near the top of the vegetation reaches a maximum value, and there is a strong shear between the top of the vegetation and the water body. This is consistent with the findings of Anjum et al. (2018) and Ghani et al. (2019b) on discontinuous bilayer patch vegetation. The Reynolds stress decreases rapidly from the top of vegetation to the water surface and is almost zero near the water surface. It also decreases rapidly from the top of the vegetation down to the inner area of the vegetation, indicating that the shear velocity of the water flow from the top to the bottom is decreasing, conducive with the deposition of sediment particles, thereby achieving the evolution of water quality, improving the water ecological environment, and weakening the sediment in the riverbed. This law is the theoretical basis explaining the dynamics of vegetation sediment deposition, and a critical factor for vegetation bank protection. Additionally, **Figure 12C, D** show that the maximum value of Reynolds stress in the submerged state increases continuously with the increase of the comprehensive stalk thickness and discrete degree of the combined vegetation. For the vegetation combination distribution (**Figure 12C**), *h* is at 0.070 m, *d*&*D* are 0.012 m & 0.015 m, 0.012 m & 0.018 m, 0.012 m & 0.021 m, and the Reynolds stress growth rates at 0.012 m & 0.012 m are 80.13%, 167.41%, and 271.56%, respectively (**Table 3**).

For the vegetation discrete distribution (**Figure 12D**), *h* is at 0.070 m, *d*&*D* are 0.009 m & 0.015 m, 0.006 m & 0.018 m, 0.003 m & 0.021 m, and the Reynolds stress growth rates at 0.012 m & 0.012 m are 80.13%, 167.41%, and 271.56%, respectively (**Table 3**). A common feature of increasing combined vegetation comprehensive stem thickness and dispersion degree is an increase of vegetation coverage. Therefore, with an increase of vegetation coverage, the maximum value of Reynolds stress also increases. This result is similar to that of Zeng and Li (2014) , who found that vegetation coverage is the key factor affecting the increase of Reynolds stress.

#### 3.4.2 Longitudinal distribution of turbulent kinetic energy in a specific longitudinal section

**Figure 13:**
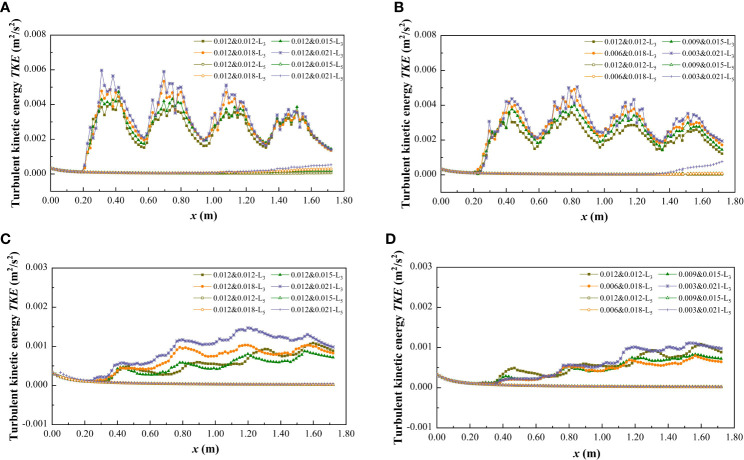
Turbulent kinetic energy distribution of longitudinal sections L_3_ and L_5_ at *h* = 0.06 m and 0.10 m under different submerged conditions. **(A)** *h* = 0.06 m, vegetation combination distribution, non-submerged vegetation, **(B)** *h* = 0.06 m, vegetation discrete distribution, non-submerged vegetation, **(C)** *h* = 0.10 m, vegetation combination distribution, submerged vegetation, **(D)** *h* = 0.10 m, vegetation discrete distribution, submerged vegetation.

Turbulent kinetic energy (TKE) is a basic parameter used to characterize the turbulent flow in natural river channels, which can directly reflect the overall turbulent flow condition. It is the average kinetic energy per unit mass related to eddy currents, and TKE is characterized by the fluctuation of root mean square velocity, which plays an important role in energy conversion and transfer. In order to clarify the influence of vegetation combination and discrete characteristics on the flow turbulence structure, the longitudinal distribution of TKE in specific longitudinal sections (L_3_ and L_5_) under different submerged conditions (*h* =0.06 m, *h* =0.10 m) is shown in **Figure 13**.

In the non-submerged state (**Figures 13A, B**), owing to the discontinuity in patch vegetation distribution, when the water flows through the patch area, the morphological resistance of the vegetation exerts a certain degree of interference, which causes the TKE in the patch area (L_3_) to be non-uniform and significantly higher than that in the non-vegetated area (L_5_). This is because the TKE value in the non-vegetated area is low and shows good uniformity due to the absence of vegetation interference. For the vegetated area (L_3_), when the water flow passes through the patch area, the TKE increases significantly and shows a zigzag distribution, and when the water flow enters the interval area, the TKE decreases significantly. This kind of patch interval distribution causes the vertical section of the vegetated area to form a turbulent region with alternating high and low. In addition, the combination form and discrete degree of vegetation are also important factors affecting the degree of water turbulence under non-submerged conditions. For the combined vegetation, the TKE increases with the increase of the comprehensive stem thickness and discrete degree of the vegetation, that is, TKE_0.012&0.012_<TKE_0.012&0.015_<TKE_0.012&0.018_<TKE_0.021&0.021_, TKE_0.012&0.012_<TKE_0.009&0.015_<TKE_0.006&0.018_<TKE_0.003&0.021_. This can be explained by the increase in the comprehensive stem thickness and the discrete degree of the combined vegetation leading to an increased vegetation coverage density. Dense vegetation provides greater resistance than sparse vegetation, causing the flow through vegetation to produce a greater turbulence intensity. The average turbulent intensity growth rate relative to a *d*&*D* of 0.012&0.012 under different combined and discrete distribution conditions is shown in **Table 4**.

**Table 4:** The average turbulent kinetic energy $\bar{TKE}$ value and its rate of change at longitudinal section L3 under different submerged states and different distribution modes.

Vegetation non-submerged state (*h*=0.06m)					
**Combined distribution *d*&*D* (m)**	$\bar{TKE}$	**Growth rate of $\bar{TKE}$ relative 0.012&0.012 (%)**	**Discrete distribution *d*&*D* (m)**	$\bar{TKE}$	**Growth rate of $\bar{TKE}$ relative 0.012&0.012 (%)**
0.012&0.012	0.00239	--	0.012&0.012	0.00239	--
0.012&0.015	0.00258	7.97%	0.009&0.015	0.00245	2.50%
0.012&0.018	0.00273	13.93%	0.006&0.018	0.00252	5.47%
0.012&0.021	0.00294	22.73%	0.003&0.021	0.00264	10.31%
**Vegetation submerged state (*h*=0.10m)**					
**Combined distribution*d*&*D* (m)**	$\bar{TKE}$	**Growth rate of $\bar{TKE}$ relative 0.012&0.012 (%)**	**Discrete distribution *d*&*D* (m)**	$\bar{TKE}$	**Growth rate of $\bar{TKE}$ relative 0.012&0.012 (%)**
0.012&0.012	0.00052	--	0.012&0.012	0.00052	--
0.012&0.015	0.00047	-10.25%	0.009&0.015	0.00045	-14.62%
0.012&0.018	0.00064	22.84%	0.006&0.018	0.00041	-21.04%
0.012&0.021	0.00086	64.90%	0.003&0.021	0.00056	6.84%

In the submerged state (**Figures 13C, D**), the variation law of the TKE value in the non-vegetated area (L_5_) was similar to that in the non-submerged state, while the TKE in the vegetated area (L_3_) no longer followed the trend of continuous increases and decreases. Instead, it gradually increased as the length of the ordinate increased, and compared with the non-submerged vegetation state, the overall TKE value in the submerged state decreased. In addition, the TKE in the vegetated area (L_3_) no longer followed the change rule that increased with the increases in comprehensive stem thickness and vegetation dispersion degree (**Table 4**), indicating that the influence of vegetation distribution pattern on flow turbulence in the free layer is weakened in the submerged state.

## 4 Conclusion

In this study, the influence of patch distribution of partial discontinuous combination vegetation on the flow characteristics of open channels was investigated using a three-dimensional Reynolds stress model. The results show that the combination and discrete distribution have significant influences on flow structure. The main conclusions are as follows:

1) For the streamwise velocity before and after introduction of a single vegetation, the diameter of the vegetation under non-submerged conditions is an important factor affecting wake structure, that is, the length of the stable wake area increases as the stem thickness increases. With the increase in stem thickness, the streamwise velocity at the trailing edge of vegetation decreased, and the difference in streamwise velocity before and after the introduction of vegetation increased with the increase in vegetation discrete degree. Under the submerged condition, the influence of vegetation distribution pattern on the streamwise velocity in the free layer is weakened.
2) For the cross-section velocity distribution, in the non-submerged state, the streamwise velocity in the non-vegetated area is significantly greater than that in the vegetated area, resulting in a strong energy exchange at the interface between the two areas. With the increase in the comprehensive stem thickness and dispersion degree of the combined vegetation, the average streamwise velocity in the vegetated area showed a decreasing trend, while that in the non-vegetated area showed an increasing trend. In the submerged state, the difference in streamwise velocity between the non-vegetated and vegetated areas decreased, and the vegetation combination and discrete distribution had no apparent effect on the difference in the streamwise velocity. In addition, the submerged state of vegetation is an important factor affecting the change in lateral velocity. In the non-submerged state, the direction of the overall lateral velocity is towards the side of the non-vegetated area, while in the submerged state, it is towards the vegetated area.
3) For the change in overall velocity along the route, as the water flows downstream, the velocity along the route in the vegetated area decreases continuously, while in the non-vegetated area it increases continuously, and the difference in velocity between the two areas becomes increasingly apparent. In addition, with the increase in comprehensive stem thickness and dispersion degree of the combined vegetation, the obstructing effect of vegetation on water flow and sediment deposition is stronger, and the water carrying capacity of the channel is also increased.
4) For the variation in Reynolds stress at the channel location of the patch area, in the non-submerged state, the Reynolds stress is highest at 1/4 of the overall water depth. In the submerged state, the Reynolds stress is highest near the top of the vegetation and there is a strong shearing effect between the top of the vegetation and the water body. With the increase in comprehensive stem thickness and the degree of dispersion of the combined vegetation, the Reynolds stress shows an increasing trend.
5) For the longitudinal turbulent kinetic energy, in the vegetated area TKE is significantly higher than that in the non-vegetated area. In the non-submerged state, the turbulence intensity in the patch area is significantly greater than that in the spaced area, forming a jagged distribution and indicating that the presence of vegetation in the patch area leads to a significant increase in the turbulent intensity of water flow. When the water flow passes through the spaced area, the turbulent kinetic energy decreases, indicating that the spaced area is a low-turbulence area, which is more suitable for the survival of aquatic organisms. The turbulent intensity of the water flow in the vegetated area increased as the comprehensive stem thickness and the discrete degree of the combined vegetation increase. In the submerged state, the turbulent kinetic energy in the vegetated area increases as the longitudinal length increases, and the influence of vegetation combination and discrete distribution on the flow turbulence in the free layer is weakened.

This study verifies that the effect of combined vegetation on the flow characteristics of open channels is different to that of uniform vegetation distribution. Therefore, it is necessary to comprehensively consider the influence of patch distribution of partial discontinuous combined vegetation on the flow characteristics of open channels, which is of great significance to the ecological application of aquatic vegetation in river channels and provides guidance for the restoration and management of eco-river systems. It should be noted that in this paper, a numerical simulation study was carried out on the flow characteristics of the longitudinally discontinuous rigid combined vegetation patches occupying one side of the river bank, and the rigid simulated vegetation was selected to focus on the study of the combined distribution and discrete distribution of the patch vegetation on the water flow structure of the river, vegetation flexibility does not take into account the effects on the flow structure,that is, it is assumed that the vegetation under the action of water flow does not deform and oscillate. However, vegetation with greater flexibility in real river courses will have a certain degree of deformation and swing under the action of water flow, which makes the water flow structure more complex. At present, the numerical model of this study does not include factors related to vegetation flexibility, so this is also the focus of our future research and the direction of our efforts.

## Data availability statement

The original contributions presented in the study are included in the article/supplementary materials. Further inquiries can be directed to the corresponding author.

## Author contributions

JZ: Conceptualization, Methodology, Writing original draft, Formal analysis, SZ: Resources, Project administration, Funding acquisition. CW: Data curation, Visualization. WW: Data curation, Visualization. LM: Supervision, Investigation. All authors contributed to the article and approved the submitted version.

## Funding

The authors acknowledge the support from the following projects: The Natural Science Foundation of Shandong Province (ZR2020ME248); Postgraduate Science and Technology Innovation Project of Shandong University of Science and Technology (YC20210826).

## Conflict of interest

The authors declare that the research was conducted in the absence of any commercial or financial relationships that could be construed as a potential conflict of interest.

## Publisher’s note

All claims expressed in this article are solely those of the authors and do not necessarily represent those of their affiliated organizations, or those of the publisher, the editors and the reviewers. Any product that may be evaluated in this article, or claim that may be made by its manufacturer, is not guaranteed or endorsed by the publisher.

## References

[B1] Aberle J. Järvelä J. (2013). Flow resistance of emergent rigid and flexible floodplain vegetation. J. Hydraul. Res. 51, 33–45. doi: 10.1080/00221686.2012.754795

[B2] Afzalimehr H. Fazel Najfabadi E. Singh V. P. (2010). Effect of vegetation on banks on distributions of velocity and reynolds stress under accelerating flow. J. Hydrol. Eng. 15, 708–713. doi: 10.1061/(ASCE)HE.1943-5584.0000229

[B3] Anjum N. Ghani U. Ahmed Pasha G. Latif A. Sultan T. Ali S. (2018). To investigate the flow structure of discontinuous vegetation patches of two vertically different layers in an open channel. Water. 10, 75. doi: 10.3390/w10010075

[B4] Anjum N. Tanaka N. (2020). Investigating the turbulent flow behaviour through partially distributed discontinuous rigid vegetation in an open channel. River. Res. Appl. 36, 1701–1716. doi: 10.1002/rra.3671

[B5] Boothroyd R. J. Hardy R. J. Warburton J. Marjoribanks T. I. (2017). Modeling complex flow structures and drag around a submerged plant of varied posture. Water. Resour. Res. 53, 2877–2901. doi: 10.1002/2016WR020186

[B6] Bordeu I. Clerc M. G. Couteron P. Lefever R. Tlidi M. (2016). Self-replication of localized vegetation patches in scarce environments. Sci. Rep-UK. 6, 1–11. doi: 10.1038/srep33703 PMC5030637 27650430

[B7] Caroppi G. Västilä K. Järvelä J. Lee C. Ji U. Kim H. S.. (2022). Flow and wake characteristics associated with riparian vegetation patches: Results from field-scale experiments. Hydrol. Process. 36, e14506. doi: 10.1002/hyp.14506

[B8] Chang W. Y. Constantinescu G. Tsai W. F. (2017). On the flow and coherent structures generated by a circular array of rigid emerged cylinders placed in an open channel with flat and deformed bed. J. Fluid. Mech. 831, 1–40. doi: 10.1017/jfm.2017.558

[B9] Cheng N. S. Hui C. L. Wang X. Tan S. K. (2019). Laboratory study of porosity effect on drag induced by circular vegetative patch. J. Eng. Mech. 145, 04019046. doi: 10.1061/(ASCE)EM.1943-7889.0001626

[B10] Cotton J. A. Wharton G. Bass J. A. B. Heppell C. M. Wotton R. S. (2006). The effects of seasonal changes to in-stream vegetation cover on patterns of flow and accumulation of sediment. Geomorphology. 77, 320–334. doi: 10.1016/j.geomorph.2006.01.010

[B11] Curran J. C. Hession W. C. (2013). Vegetative impacts on hydraulics and sediment processes across the fluvial system. J. Hydrol. 505, 364–376. doi: 10.1016/j.jhydrol.2013.10.013

[B12] Dey S. (2014). “Turbulence in open-channel flows,” in Fluvial hydrodynamics (Berlin, Heidelberg: Springer), 95–187.

[B13] Fathi-Moghadam M. Kashefipour M. Ebrahimi N. Emamgholizadeh S. (2011). Physical and numerical modeling of submerged vegetation roughness in rivers and flood plains. J. Hydrol. Eng. 16, 858–864. doi: 10.1061/(ASCE)HE.1943-5584.0000381

[B14] Folkard A. M. Gascoigne J. C. (2009). Hydrodynamics of discontinuous mussel beds: Laboratory flume simulations. J. Sea. Res. 62, 250–257. doi: 10.1016/j.seares.2009.06.001

[B15] Ghani U. Anijum N. Pasha G. A. Ahmad M. (2019a). Investigating the turbulent flow characteristics in an open channel with staggered vegetation patches. River. Res. Appl. 35, 966–978. doi: 10.1002/rra.3460

[B16] Ghani U. Anjum N. Pasha G. A. Ahmad M. (2019b). Numerical investigation of the flow characteristics through discontinuous and layered vegetation patches of finite width in an open channel. Environ. Fluid. Mech. 19, 1469–1495. doi: 10.1007/s10652-019-09669-x

[B17] Huai W. X. Zhang J. Wang W. J. Katul G. G. (2019). Turbulence structure in open channel flow with partially covered artificial emergent vegetation. J. Hydrol. 573, 180–193. doi: 10.1016/j.jhydrol.2019.03.071

[B18] Jang C. L. Shimizu Y. (2007). Vegetation effects on the morphological behavior of alluvial channels. J. Hydraul. Res. 45, 763–772. doi: 10.1080/00221686.2007.9521814

[B19] Järvelä J. (2002). Flow resistance of flexible and stiff vegetation: A flume study with natural plants. J. Hydrol. 269, 44–54. doi: 10.1016/S0022-1694(02)00193-2

[B20] Kazem M. Afzalimehr H. Sui J. (2021a). Formation of coherent flow structures beyond vegetation patches in channel. Water. 13, 2812–2827. doi: 10.3390/w13202812

[B21] Kazem M. Afzalimehr H. Sui J. (2021b). Characteristics of turbulence in the downstream region of a vegetation patch. Water. 13, 3468–3488. doi: 10.3390/w13233468

[B22] Kemp J. L. Harper D. M. Crosa G. A. (2000). The habitat-scale ecohydraulics of rivers. Ecol. Eng. 16, 17–29. doi: 10.1016/S0925-8574(00)00073-2

[B23] Li W. Q. Dan W. Jiao J. L. Yang K. J. (2018). Effects of vegetation patch density on flow velocity characteristics in an open channel. J. Hydrodyn. 31, 1052–1059. doi: 10.1007/s42241-018-0086-6

[B24] Li D. Huai W. X. Liu M. Y. (2020). Investigation of the flow characteristics with one-line emergent canopy patches in open channel. J. Hydrol. 590, 125248. doi: 10.1016/j.jhydrol.2020.125248

[B25] Liu D. Diplas P. Hodges C. C. Fairbanks J. D. (2010). Hydrodynamics of flow through double layer rigid vegetation. Geomorphology 116, 286–296. doi: 10.1016/j.geomorph.2009.11.024

[B26] Liu X. D. Liu Z. Q. Tang L. C. Han Y. Yang S. Q. (2021). Analysis of vegetation resistance based on two typical distribution types in ecological channel. Ecol. Eng. 169, 106325. doi: 10.1016/j.ecoleng.2021.106325

[B27] Meire D. Kondziolka J. Nepf H. (2014). Interaction between neighboring vegetation patches: impact on flow and deposition. Water. Resour. Res. 50, 3809–3825. doi: 10.1002/2013WR015070

[B28] Mulahasan S. Stoesser T. (2017). Flow resistance of in-line vegetation in open channel flow. Int. J. River. Basin Manage. 15, 329–334. doi: 10.1080/15715124.2017.1307847

[B29] Nadaoka K. Yagi H. (1998). Shallow-water turbulence modelling and horizontal large-eddy computation of river fow. ASCE. J. Hydraul. Eng. 124, 493–500. doi: 10.1061/(ASCE)0733-9429(1998)124:5(493

[B30] Nept H. M. (2012). Hydrodynamics of vegetated channels. J. Hydraul. Res. 50, 262–279. doi: 10.10.80/00221686.2012.696559

[B31] Nicolle A. Eames I. (2011). Numerical study of flow through and around a circular array of cylinders. J. Fluid. Mech. 679, 1–31. doi: 10.1017/jfm.2011.77

[B32] Nosrati K. Afzalimehr H. Sui J. (2022). Drag coefficient of submerged flexible vegetation patches in gravel bed rivers. Water. 14, 743. doi: 10.3390/w14050743

[B33] Rominger J. T. Nepf H. M. (2011). Flow adjustment and interior flow associated with a rectangular porous obstruction. J. Fluid. Mech. 680, 636–659. doi: 10.1017/jfm.2011.199

[B34] Ruiz-Reynés D. Gomila D. Sintes T. Hernández-García E. Marbà N. Duarte C. M. (2017). Fairy circle landscapes under the sea. Sci. Adv. 3, e1603262. doi: 10.1126/sciadv.1603262 28782035 PMC5540242

[B35] Sand Jensen K. Madsen T. V. (1992). Patch dynamics of the stream macrophyte, callitriche cophocarpa. Freshw. Biol. 27, 277–282. doi: 10.1111/j.1365-2427.1992.tb00539.x

[B36] Schulz M. Kozerski H. P. Pluntke T. Rinke K. (2003). The influence of macrophytes on sedimentation and nutrient retention in the lower river spree (germany). Water. Res. 37, 569–578. doi: 10.1016/S0043-1354(02)00276-2 12688691

[B37] Shi Y. Jiang B. Nepf H. M. (2016). Influence of particle size and density, and channel velocity on the deposition patterns around a circular patch of model emergent vegetation. Water. Resou. Res. 52, 1044–1055. doi: 10.1002/2015WR018278

[B38] Takemura T. Tanaka N. (2007). Flow structures and drag characteristics of a colony-type emergent roughness model mounted on a flat plate in uniform flow. Fluid. Dyn. Res. 39, 694–710. doi: 10.1016/j.fluiddyn.2007.06.001

[B39] Tanaka T. Ohmoto T. (2015). Turbulent structure in open channel with permeable and impermeable side cavities. J. Appl. Water. Eng. Res. 4, 44–53. doi: 10.2208/jscejhe.68.I_805

[B40] Tanaka T. Ohmoto T. Tanaka T. (2008). Flow resistance and momentum transport in open channel with longitudinaly discontinuous vegetation. Proceed. Hydraul. Eng. 52, 763–768. doi: 10.2208/prohe.52.763

[B41] Tang X. Rahimi H. Guan Y. Wang Y. (2021). Hydraulic characteristics of open-channel flow with partially-placed double layer rigid vegetation. Environ. Fluid. Mech. 21, 317–342. doi: 10.1007/s10652-020-09775-1

[B42] Tang C. Yi Y. Jia W. Zhang S. (2020). Velocity and turbulence evolution in a flexible vegetation canopy in open channel flows. J. Clean. Prod. 270, 122543. doi: 10.1016/j.jclepro.2020.122543

[B43] Tanino Y. Nepf H. M. (2008). Laboratory investigation of mean drag in a random array of rigid, emergent cylinders. J. Hydraul. Eng. 134, 34–41. doi: 10.1061/(ASCE)0733-9429(2008)134:1(34

[B44] Tny A. Phsdl A. Dfs A. Cgdap A. Jgja B. Hmn B. (2019). From patch to channel scale: the evolution of emergent vegetation in a channel. Adv. Water. Resour. 129, 131–145. doi: 10.1016/j.advwatres.2019.05.009

[B45] Tooth S. Nanson G. C. (2000). The role of vegetation in the formation of anabranching channels in an ephemeral river, northern plains, arid central Australia. Hydrol. Process. 14, 3099–3117. doi: 10.1002/1099-1085(200011/12)14:16/17<3099::AID-HYP136>3.0.CO;2-4

[B46] Truong S. H. Uijttewaal W. S. J. (2019). Transverse momentum exchange induced by large coherent structures in a vegetated compound channel. Water Resour. Res. 55, 589–612. doi: 10.1029/2018WR023273

[B47] Vandenbruwaene W. Temmerman S. Bouma T. J. Klaassen P. C. de Vries M. B. Callaghan D. P.. (2011). Flow interaction with dynamic vegetation patches: implications for biogeomorphic evolution of a tidal landscape. J. Geophys. Res.-Earth Surf. 116, F01008. doi: 10.1029/2010JF001788

[B48] Velísková Y. Dulovičová R. Schügerl R. (2017). Impact of vegetation on flow in a lowland stream during the growing season. Biologia. 72, 840–846. doi: 10.1515/biolog-2017-0095

[B49] Wang M. Avital E. Chen Q. Williams J. Xie Q. (2021). A numerical study on suspended sediment transport in a partially vegetated channel flow. J. Hydro. 599, 126335. doi: 10.1016/j.jhydrol.2021.126335

[B50] White B. L. Nepf H. M. (2007). Shear instability and coherent structures in shallow flow adjacent to a porous layer. J. Fluid. Mech. 593, 1–32. doi: 10.1017/S0022112007008415

[B51] White B. L. Nepf H. M. (2008). A vortex-based model of velocity and shear stress in a partially vegetated shallow channel. Water. Resou. Res. 44, W01412. doi: 10.1029/2006WR005651

[B52] Widdows J. Navarro J. M. (2007). Influence of current speed on clearance rate, algal cell depletion in the water column and resuspension of biodeposits of cockles (Cerastoderma edule). J. Exp. Mar. Biol. Ecol. 343, 44–51. doi: 10.1016/j.jembe.2006.11.011

[B53] Wu F. S. Wang W. Y. Jiang S. H. (2007). Development of hydrodynamics in vegetated open channel. Adv. Water. Sci. 18, 456–461. doi: 10.14042/j.cnki.32.1309.2007.03.024

[B54] Yang Y. Irish J. L. Socolofsky S. A. (2015). Numerical investigation of wave-induced flow in mound-channel wetland systems. Coast. Eng. 102, 1–12. doi: 10.1016/j.coastaleng.2015.05.002

[B55] Yang D. Xiong D. Zhang B. Guo M. Su Z. Dong Y.. (2017). Effect of grass basal diameter on hydraulic properties and sediment yield processes in gully beds in the dry-hot valley region of southwest China. Catena. 152, 299–310. doi: 10.1016/j.catena.2017.01.023

[B56] Zdankus N. Punys P. Martinaitis E. Zdankus T. (2016). Lowland river flow control by an artificial water plant system. River. Res. Appl. 32, 1382–1391. doi: 10.1002/rra.2973

[B57] Zeng C. Li C. W. (2014). Measurements and modeling of open-channel flows with finite semi-rigid vegetation patches. Environ. Fluid. Mech. 14, 113–134. doi: 10.1007/s10652-013-9298-z

[B58] Zhang J. Su X. (2008). Numerical model for flow motion with vegetation. J. Hydrodyn. 20, 172–178. doi: 10.1016/S1001-6058(08)60043-8

[B59] Zhang S. T. Wang Z. K. Liu Y. Li G. B. Chen S. Liu M. (2020). The influence of combined vegetation stalk thickness on water flow resistance. Water. Environ. J. 34, 455–463. doi: 10.1111/wej.12480

[B60] Zhao F. Huai W. (2016). Hydrodynamics of discontinuous rigid submerged vegetation patches in open-channel flow. J. Hydro-environ. Res. 12, 148–160. doi: 10.1016/j.jher.2016.05.004

